# Circulating miRNA Signaling for Fatty Acid Metabolism in Response to a Maximum Endurance Test in Elite Long-Distance Runners

**DOI:** 10.3390/genes15081088

**Published:** 2024-08-17

**Authors:** Dailson Paulucio, Carlos Ramirez-Sanchez, Rodolfo Velasque, Raphael Xavier, Gustavo Monnerat, Adrieli Dill, Juliano Silveira, Gabriella M. Andrade, Flavio Meirelles, Marcos Dornelas-Ribeiro, Benedikt Kirchner, Michael W. Pfaffl, Fernando Pompeu, Caleb G. M. Santos

**Affiliations:** 1 Biometrics Laboratory (LADEBIO), Federal University of Rio de Janeiro, Rio de Janeiro 21941-599, Brazil; dailsonpaulucio@gmail.com (D.P.);; 2 Instituto de Biofisica Carlos Chagas Filho, Universidade Federal do Rio de Janeiro, Rio de Janeiro 21941-170, Brazil; 3 Brazilian Army Institute of Biology, Research, Teaching and Research Division, Rio de Janeiro 20911-270, Brazil; 4 Faculty of Animal Sciences and Food Engineering, Universidade de São Paulo, Pirassununga 13635-900, Brazil; 5 Department of Animal Physiology and Immunology, School of Life Sciences, Technical University of Munich, Liesel-Beckmann-Straße 1, 85354 Freising, Germany

**Keywords:** miRNA, maximal oxygen uptake, exercise performance, endurance elite athletes, fatty acid metabolism

## Abstract

Maximal oxygen uptake (VO_2_max) is a determining indicator for cardiorespiratory capacity in endurance athletes, and epigenetics is crucial in its levels and variability. This initial study examined a broad plasma miRNA profile of twenty-three trained elite endurance athletes with similar training volumes but different VO_2_max in response to an acute maximal graded endurance test. Six were clustered as higher/lower levels based on their VO_2_max (75.4 ± 0.9 and 60.1 ± 5.0 mL.kg^−1^.min^−1^). Plasma was obtained from athletes before and after the test and 15 ng of total RNA was extracted and detected using an SYBR-based 1113 miRNA RT-qPCR panel. A total of 51 miRNAs were differentially expressed among group comparisons. Relative amounts of miRNA showed a clustering behavior among groups regarding distinct performance/time points. Significantly expressed miRNAs were used to perform functional bioinformatic analysis (DIANA tools). Fatty acid metabolism pathways were strongly targeted for the significantly different miRNAs in all performance groups and time points (*p* < 0.001). Although this pathway does not solely determine endurance performance, their significant contribution is certainly achieved through the involvement of miRNAs. A highly genetically dependent gold standard variable for performance evaluation in a homogeneous group of elite athletes allowed genetic/epigenetic aspects related to fatty acid pathways to emerge.

## 1. Introduction

Maximal oxygen uptake (VO_2_max) is a parameter of cardiorespiratory capacity, a crucial indicator of good health, and a determining factor for endurance performance [1,2]. Notably, heredity may play a significant role in determining the response of VO_2_max to training, accounting for up to 47% of the variability [3]. Advancements in molecular technologies that can analyze larger amounts of data have shown promise in enhancing our understanding of molecular trainability and endurance adaptations [4,5]. But physiological adaptation requires altering the expression of available genes and enzymes, which relies on epigenetic mechanisms that depend, in turn, on the expression of RNAs.

Transcribed by RNApol II as a small (~22 nt) and non-coding RNA, the 2654 miRNAs described in humans [6] play a crucial role in modulating gene expression for physiological responses. Although the DNA sequences that give rise to them comprise between 1 and 5% of the genome, miRNA can regulate up to 30% of protein-coding gene expression [7]. Therefore, the expression profiles of serum/plasma miRNAs and their stability can signal essential cellular and tissue functions, making these molecules important candidates for biomarkers, not only related to pathologies or injuries but also associated with the physiological adaptations promoted by exercise, and finally, endurance performance [8,9,10,11]. The inhibitory mechanism of miRNA on mRNA expression may affect several biological processes essential for exercise, including angiogenesis, cardiac and skeletal muscle contraction, energy hypertrophy, inflammation, and mitochondrial metabolism [12,13,14].

For example, in the acute exercise of elite athletes, epigenetics is likely to contribute to both performance and recovery. This is because the highest endurance performance depends on the ability to sustain physical activity for a more extended period while maintaining stable systemic glucose levels. For this, the oxidation of fatty acids during submaximal and maximal intensity exercises is crucial and can provide a competitive advantage. Furthermore, lipids are known to configure the primary fuel during recovery from any activity, which results in glycogen depletion. Despite significant advancements in our understanding of circulating biomarkers, there is a notable gap in the literature regarding the role of miRNAs in response to acute exercise among elite endurance athletes [15]. These athletes exhibit unique and remarkable physiological adaptations that enable them to perform exceptionally. This initial study aimed to examine the plasma miRNA profiles of elite high-performance endurance athletes with similar training volumes but different levels of VO_2_max in response to an acute maximal graded endurance test (GXT).

## 2. Materials and Methods

### 2.1. Study Design

Twenty-three Brazilian elite male long-distance runners, endurance specialists, were selected for a cross-sectional study with two visits at intervals of 48 h/15 days (Supp. methods, Figure S1). They had to present at least 100 km/week of training volume, two years of experience, 18 years of age, and 500 points in the World Athletics ranking. The first visit included a medical and anthropometric evaluation in which individuals with recent orthopedic injuries or using medications/supplements that could have affected physical performance were excluded. During the second visit, at rest after eight hours of fasting, a blood sample was obtained (PRE). Afterward, 60 min after a standardized breakfast, the subject performed an ergometric protocol based on a GXT on a treadmill to determine VO_2_max. The Supplementary Methods describe the protocol in detail. Immediately after the GXT, a new blood sample was obtained (POST). The study was approved by the local Ethics Committee (59983516.0.0000.5257). Written informed consent approval was prospectively obtained for all subjects. Patients and the public were not involved in this research’s design, conduct, reporting, or dissemination plans.

### 2.2. Molecular Analysis

Peripheric blood was collected in 4 mL K2EDTA tubes to obtain plasma (3000× *g*; 12 min) and stored at −80 °C. Total RNA was obtained from 200 µL of plasma following the manufacturer’s protocol of miRNeasy Serum/Plasma Kit (Qiagen, Hilden, Germany), and it was evaluated using the spectrophotometer NanoDrop 2000 (Thermo Fisher Scientific, Waltham, MA, USA). cDNA synthesis, and subsequently, the detection of 1113 miRNAs was performed starting from 15 ng of total RNA using the SYBR-based RT-qPCR panel hsa-miRNome miRNA Profiling Kit (System Biosciences, Mountain View, CA, USA) following the manufacturer’s protocol in QuantStudio 5™ Real-Time System (Thermo Fisher Scientific, Waltham, MA, USA). The non-template control (NTC), calibrators, and stably expressed housekeeping candidate genes (U6 snRNA, RNU43 snoRNA, U1 snRNA) were evaluated. Overall quality control of the RT-qPCR and calculation of the relative gene expression values were performed by the GenEx software v 7.1.1.118 (MultiD Analyses, Göteborg, Sweden), strictly following the MIQE guidelines [16] (see Supplementary Material Figure S2).

### 2.3. Statistical Analysis

Physical-based variables: The Shapiro–Wilk test was used to verify the distribution, and Levene’s test was used to verify homoscedasticity. Descriptive statistics included mean and standard deviation. The significance level was α ≤ 0.05. Different performance groups were compared using a *t*-test for independent samples. 

**Figure 1:**
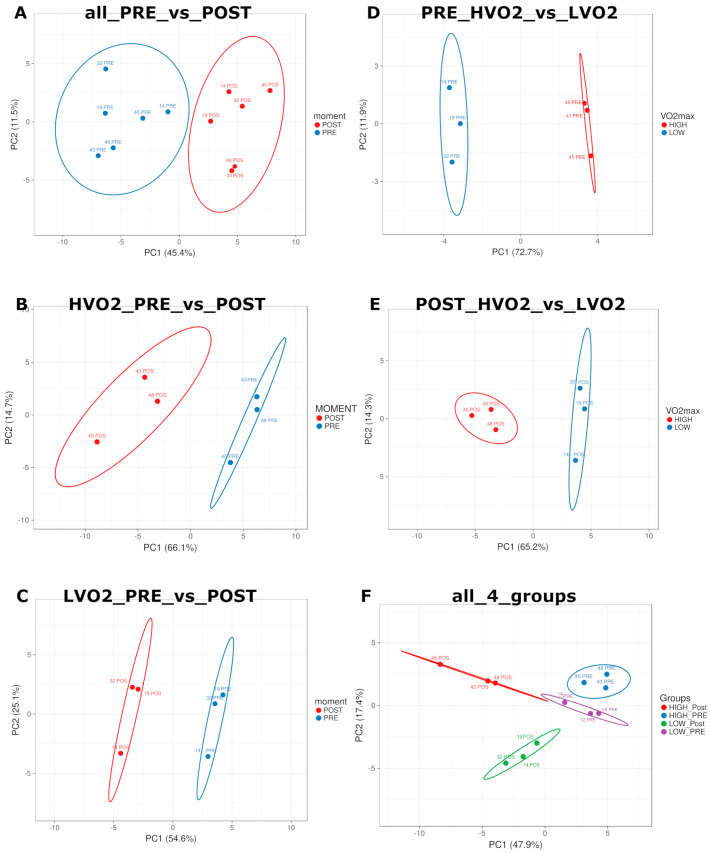
Principal component analysis (PCA) graphs showing “timepoint”−based clustering analysis between (**A**) all_PRE_vs_POST, (**B**) HVO2_PRE_vs_POST, and (**C**) LVO2_PRE_vs_POST comparisons; additional PCA showing “performance”−based clustering analysis between (**D**) PRE_HVO2_vs_LVO2 and (**E**) POST_HVO2_vs_LVO2. And finally, a global clustering analysis between (**F**) all_4_groups. PCA was performed using the relative amount of RNAs significantly detected among groups. Confident intervals are presented inside every colored ellipse.

miRNA-related variables: The Shapiro–Wilk test was applied to check for normal data distribution. The relative expression of miRNA was compared using a two-tailed non-paired *t*-test for “performance” comparisons regarding higher VO_2_max (HVO2) and lower VO_2_max (LVO2) (PRE_HVO2_vs_LVO2, POST_HVO2_vs_LVO2); a two-tailed paired *t*-test for “timepoint” comparisons PRE and POST exercise (all_PRE_vs_POST, LVO2_PRE_vs_POST, HVO2_PRE_vs_POST); and a one-way ANOVA for “global” comparison (all_4_groups). To prevent a false discovery rate due to multiple tests, the Benjamini–Hochberg correction was applied. Using the whole dataset first (Supp. results, Figure S3), and subsequently, the miRNAs significantly (*p* < 0.05) detected among the groups (Figure 1), distance matrix and principal component analysis (PCA) were performed using the ClustVis tool (available at: biit.cs.ut.ee/clustvis/—accessed on 20 May 2023).

### 2.4. Functional Bioinformatic Analysis

The miRNA nomenclature was adjusted and validated according to version 22.1 of miRbase (Supp. results, Tables S1 and S2), using the original primers of every miRNA detected. Functional bioinformatics analysis (FBA) was performed using the mirPath (v.3.0) tool and Tarbase v7.0 from the DIANA tools [17]. FBA was performed to obtain the core pathways affected in every comparison using up to the 20 best-ranked miRNAs significantly differently regulated based on *p*-value, excluding those with log2fold differences between −1 and 1. Pathways obtained individually were merged using the “pathways union” mode for enrichment analysis and heatmap construction based on Fisher’s exact test for hypergeometric distribution and false discovery rate (FDR) correction. The KEGG database was used for pathway denomination/grouping and heatmap performing. An additional EASE score (modified Fisher’s exact test) was applied to the results for an even more conservative and rigorous statistical analysis.

## 3. Results

### 3.1. Physical Performance-Related Variables

Based on decreasing VO_2_max levels, athletes were ranked and divided into tertiles (8, 7, and 8 subjects) (supp. results, Figure S4). So, we selected six unique endurance athletes: three samples from the first and three from the third tertiles. The total RNA levels were clustered in to two performance groups: HVO2 and LVO2. Both groups were homogeneous for all variables related to physical performance but clearly had different cardiorespiratory capacities based on their VO_2_max levels (Table 1). HVO2 presented at least 15 mL/kg/min more than the LVO2 group (25%). The training volume was compatible with an elite international performance athlete, and their training experience points for some level of endurance chronic adaptation.

### 3.2. miRNA Profile in Response to Acute Exercise

**Figure 2:**
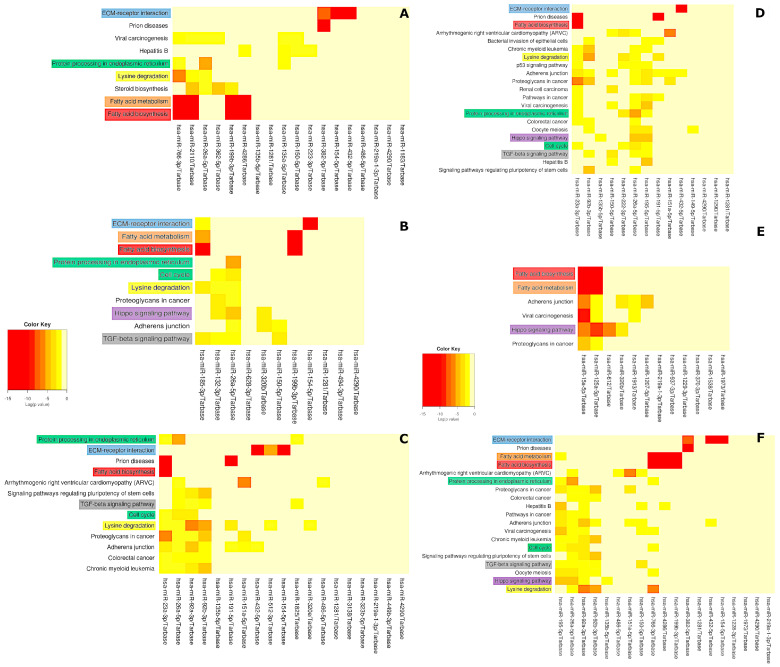
Heatmaps of significant pathways (Fishers’ exact test and false discovery ratio (FDR) correction) related to crucial miRNAs in response to a maximal graded endurance test (GXT) (timepoint). (**A**) All athletes (all_PRE_vs_POST); (**B**) LVO2_PRE_vs_POST; (**C**) HVO2_PRE_vs_POST; (**D**) PRE_HVO2_vs_LVO2); (**E**) POST_HVO2_vs_LVO2; (**F**) all_4_groups (HVO2_PRE vs. HVO2_POST vs. LVO2_PRE vs. LVO2_POST). Pathways highlighted in red represent fatty acid biosynthesis; in orange, fatty acid metabolism; in purple, Hippo signaling; in gray, TGF-β; in yellow, lysine degradation; and in green, other related pathways.

Initially, we analyzed the relative quantities of plasma miRNAs for acute exercise impact (all_PRE_vs_POST) independently of performance (n = 6). Using PCA on the whole dataset (Supp. results, Figure S3) and on all miRNAs significantly different between groups (paired analysis), we observed a clustering behavior (Figure 1A). An intragroup PCA clustering was observed in HVO2_PRE_vs_POST, even with only three individuals per group (Figure 1B). Similarly, in LVO2_PRE_vs_POST clustering was apparent (Figure 1C). The essential miRNAs for every clustering analysis are summarized in Table 2, statistically detailed in Table S3 (Supp. results), and intersected to the pathway related in heatmaps in Figure 2.

### 3.3. miRNA Profile Related to Each Group’s Performance

Both “PRE” and “POST” miRNAs (non-paired analysis) were able to show a PCA clustering between the HVO2 and LVO2 groups based on exercise performance (Figure 1D,E). No clear clustering was observed between the four groups together (Figure 1F), but the ANOVA analysis did not consider paired statistics between “time points”. Important miRNAs for clustering are summarized in Table 2 and statistically detailed in Table S4 (supp. results).

### 3.4. Functional Bioinformatic Analysis

Essential miRNAs for every comparison were simultaneously used to obtain their predicted or experimentally tested target genes and related pathways (Figure 2). The heatmaps below show the significant pathways (corrected for FDR) for each comparison. They present 9 significant pathways for the all_PRE_vs_POST comparison, 10 significant pathways for LVO2_PRE_vs_POST, and 13 significant pathways for HVO2_PRE_vs_POST (Figure 2A–C). Including performance evaluation, 21 pathways were important to cluster PRE_HVO2_vs_LVO2, 6 for POST_HVO2_vs_LVO2, and 19 for all_4_groups (Figure 2D–F). In general, pathways related to fatty acid biosynthesis/metabolism were observed in all possible comparisons. Other pathways strongly associated with miRNAs included extracellular matrix (ECM)–receptor interaction, lysine degradation, Hippo signaling, and transforming growth factor (TGF-β). In Table 3, only the most strongly associated pathways after the results underwent the EASE score conservative statistical correction are presented. The pathways most likely to be related to acute exercise responses and performance are highlighted in color (Figure 2, Table 3).

## 4. Discussion

### 4.1. miRNAs and Elite Endurance Athletes

The 3′UTR regulatory, inhibitory transcription mechanism of miRNAs engages some pathways mainly because of their ability to target a diverse range of genes. However, the regulated genes may, in turn, have stimulatory or inhibiting roles concerning a high-intensity endurance activity, for example. Therefore, the miRNA expression profile may contribute to elucidating essential mechanisms associated with high performance [18] or even be a promising approach toward developing individualized training strategies [11].

Detecting miRNAs before exercise reflects an athlete’s resting physiological state, influenced by their chronic endurance adaptation. Conversely, detecting molecules immediately after exercise may show variations caused by cell lysis due to micro-injuries resulting from intense physical activity, making some of them more readily available in the plasma [19]. After the GXT (usually 20–30 min), plasma could also capture initial cellular responses to sustained maximum effort or even to signal the post-exercise period as the test’s conclusion draws near. It is known that transient post-exercise changes include transcription of myogenic regulators, carbohydrate and lipid metabolism-/mobilization-, and mitochondrial metabolism-related genes [20]. Moreover, we need to highlight that skeletal muscles are the largest organ in the body [21]. In response to a GXT, the pool of plasma RNAs may originate from already-circulating RNAs, added to new ones primarily from muscle, vascular, or blood cells, associated or not with apoptotic bodies, protein complexes, extracellular vesicles, or lipoproteins [22,23]. Examining the miRNAs that comprise each fraction may provide even more valuable insights [24].

Some detected miRNAs important for several groups or time points could hypothetically belong to vital physiological pathways related to exercise or indicate an essential degree of evolutionary conservation, as they generally appear, often irrespective of the group or moment, in our homogeneous population [25]. In our study, miRNAs such as miR-1281, miR-150-5p, miR-26a-5p, miR-4290, and miR-199b-3p were differently detected in several of the comparisons (Table 2, Figure S5/Supp. results). miR-199b-3p and miR-150-5p, for example, played a key role in clustering for all PRE_vs_POST exercise comparisons, with the latter being detected at higher levels in plasma after 10 km of running in athletes previously [26].

Particularly, recent results connected the mir-199-3p family to the conversion of slow to fast muscle fibers and muscle regeneration in mice [27,28], IGF1/Akt/mTOR pathways, muscle regeneration in human cells, and fatty acid metabolism [29]. Additionally, the mir-199-3p family was related to pathological cardiac hypertrophy and even physiological cardiac hypertrophy with increased stroke volume/VO_2_max, typical of endurance adaptation training [30]. On the other hand, miRNA unique to specific groups could indicate an influence on specific pathways related to acute exercise or chronic adaptations [31]. mir-15a-5p and miR-1538, for example, were important for performance clustering only for POST_HVO2_vs_LVO2, while hsa-miR-195-5p and miR-1290 for PRE_HVO2_vs_LVO2; or even miR-486-5p for the HVO2_PRE_vs_POST comparison (Table 2, Figure S5/Supp. results). Corroborating our study, miR-486-5p was previously downregulated in athletes’ plasma after exercise and was also related to higher VO_2_max levels [32]. 

### 4.2. Endurance Adaptation, Functional Analysis, and Fatty Acid-Related Pathways

The need for more profound studies in elite athletes makes our results useful for highly adapted athletes and, to some extent, a possible target profile of plasma transcripts for developing athletes. miRNAs are differentially expressed according to the type, intensity, level of adaptation, and exercise volume [33]. Consequently, a valid biomarker proposal must begin from an imperatively uniform selection of subjects. All the selected athletes were homogeneously well-trained and experienced endurance runners (Table 1), chosen from 23 ranked athletes according to VO_2_max levels. This selection is crucial because managing energy stores is critical during endurance exercises. A shift in substrate utilization from glucose to fat is a hallmark of an endurance-trained muscle [34].

In addition, elite athletes adapted to endurance have a higher proportion of type I oxidative slow-twitch fibers, and a higher capillary-to-fiber ratio and mitochondrial volume density [35]. In turn, these fibers have a higher capacity for fatty acid metabolism and biosynthesis, contributing to maintaining physical activity for a more extended period while preserving systemic glucose levels [36]. Interestingly, the enzyme 3-hydroxyacyl-CoA-dehydrogenase (HADHA), involved in fatty acid oxidation, has 20% higher activity in skeletal muscle fibers from Kenyans, a dominant ethnicity among elite long-distance runners [36,37,38]. Specific populations from Kenya and Ethiopia, even more associated with endurance performance, had lipid metabolism-enriched enriched gene sets compared to close populations [36].

Most of the critical miRNAs related to fatty acid biosynthesis identified in our analysis were functionally associated with core enzymes: Acyl-CoA synthetase 4 (ACSL4), acetyl-CoA carboxylase alpha (ACACA), but mainly to fatty acid synthase (FASN). FASN plays a crucial role in all phases of fatty acid biosynthesis, including initiation, elongation, and even in the mitochondrial step (Figure S6). FBA analysis showed that FASN is not only targeted primarily by miR-199b-3p but also by miR-4286, miR-766-3p, miR-2110, miR-185-3p, miR-23a-3p, miR-15a-5p, and miR-125a-5p. In our data, most of those miRNAs related to FASN were less detected after exercise (Supp. results, Table S3). This points to a release signal for FASN and related enzymes acting for fatty acid recovery after exercise. FASN, more available after exercise, is related to endurance performance due to fatty acids’ ability to maintain endurance exercise. But the same miR-766-3p, miR-15a-5p, and additionally miR-195-5p also target HADHA, involved in fatty acid oxidation. Conversely, in POST_HVO2_vs_LVO2, miR-15a-5p levels were 23-fold higher in LVO2 (4.5 log2fold) (Supp. results, Table S4), and the classical understanding is that lipids become the predominant fuel during recovery from exercise that results in glycogen depletion [15]. So, higher levels of HADHA in elite athletes could result in better fatty acid oxidation and endurance performance. Our study in elite athletes showed fatty acid-related pathways do not solely determine endurance performance; however, their significant contribution is certainly achieved through the involvement of miRNAs.

#### Other Associated Pathways

The Hippo signaling pathway was essential for clustering in LVO2_PRE_vs_POST and for the PRE_HVO2_vs_LVO2 and POST_HVO2_vs_LVO2 performance-based comparisons (Table 3). Different miRNAs engage the pathway depending on the timepoint: PRE (miR-26a-5p, miR-195-5p, and miR-135b-5p) or POST (miR-15a-5p, miR-125a-5p, miR-320b, miR-612). As previously described, mechanic signals from endurance exercise inhibit the Hippo pathway, releasing transcriptional coactivators (Yes-associated protein—YAP; and transcriptional coactivator with PDZ-binding motif—TAZ) to activate genes involved in the cell cycle and proliferation, crucial steps for tissue regeneration [39,40,41]. Among more than 50 target genes (Table 3), we highlighted specific kinases (LATS1/2) commonly described in the literature [42,43] and targeted by our miRNAs. This suggests their importance for clustering groups and exercise regulation in our endurance athletes via Hippo pathways. However, the diversity of plasma levels of miRNAs between groups (Supp results, Tables S3 and S4) and their regulation of multiple genes points to this pathway’s role in fine physiological adjustment via miRNAs.

In the HVO2_PRE_vs_POST, PRE_HVO2_vs_LVO2, and all_4_groups comparisons, five significant miRNAs for group clustering targeted at least 18 different genes related to lysine degradation (Table 3). Obviously, after a GXT we can expect biomarkers reflecting heightened utilization of several fuel substrates, including amino acid catabolism. Lysine degradation leads to the final formation of carnitine or acetyl-CoA, a core molecule for the tricarboxylic acid (TCA) cycle and ATP production. Previous metabolomic data showed lower plasma levels of lysine and higher levels of TCA intermediates after a marathon [31]. Our FBA for lysine degradation showed that most of our related miRNAs are present in greater amounts before exercise, mainly the mir-92 family (Supp. results, Table S3). This family has at least 11 target genes directly involved in the degradation of lysine-containing proteins and the KMT2E gene, which promotes the conversion of acetoacetyl-CoA into acetyl-CoA. Although the trained subjects had lower mir-92a-3p levels [44], our athletes had even less of this miRNA after exercise, signaling the need to activate the lysine degradation pathway after exercise (Supp. results, Table S3).

TGF-β is an important regulator of muscle growth and repair. Often released during the anti-inflammatory phase of leukocyte polarization, it is vital to produce matrix proteins and remodel the ECM to accommodate repaired and novel myofibers after extensive exercise or during endurance adaptation [35,45]. Training-response transcriptomic data showed that high-responders to endurance training had tissue remodeling pathways regulated by TGF-β as a central feature of their phenotype [46]. Previously, using a smaller PCR panel of 72 miRNAs, amateur ultramarathon runners’ plasma showed TGF-β as a crucial enriched pathway 30 min after running 100 km, but via different miRNAs from our analysis. Important for the LVO2_PRE_vs_POST and PRE_HVO2_vs_LVO2 comparisons (Table 2), miR-185-3p, miR-26a-5p, miR-150-5p, miR-132-3p, miR-195-5p, and miR-92b-3p targeted TGF-β signaling through at least 34 related genes. Although important, the diversity in plasma miRNA levels between groups (Supp results, Tables S3 and S4), targeting an even more significant number of genes, limited our ability to gain a unified understanding of TGF-β′s role via miRNAs in our groups.

Five significant miRNAs (miR-432-5p, miR-154-5p, miR-382-5p, miR-512-3p, miR-185-3p) targeted at least 11 different genes of ECM–receptor-related pathways in all comparisons excepting POST_HVO2_vs_LVO2. Corroborating, our data showed consistently higher miRNA ECM-related levels before exercise (all_PRE_vs_POST, LVO2_PRE_vs_POST, HVO2_PRE_vs_POST) or in the LVO2 group (PRE_HVO2_vs_LVO2) (Supp. results, Tables S3 and S4). Recently, robust proteomic data showed that most ECM-related proteins increased in plasma after exercise [47,48]. In muscle tissue, a study reported that ECM-related genes were upregulated in sedentary [49] and active subjects after acute endurance exercise, in a consistent metanalysis with more than 66 published datasets [47]. Accordingly, our data revealed a distinct amount of plasma miRNAs targeting genes related to the ECM pathway, which supports the idea of muscles requiring ECM remodeling for post-exercise recovery. Furthermore, this may suggest that high-performance athletes may be better equipped to perform such remodeling.

### 4.3. Multifactorial Traits Challenge

It is important to highlight that in multifactorial traits that are genetically dependent, the individual contribution of genes is usually small. Still, the combination of them can provide a more reliable understanding [50]. Elite performance clearly depends on training-related improvements in physiological/biochemical processes and genetic/inherited factors. Logically, miRNA expression or their plasma availability cannot explain all epigenetic variability, but they appear to explain these factors very well [2]. This work provided an important screening and unique insight to improve the knowledge of acute exercise mechanisms and to understand more about biomarkers or transcriptomic predictors of elite athletes’ cardiorespiratory capacity using serum miRNAs. The most significant miRNAs for each comparison, like in higher-performance athletes, can be a kind of serum “target” for other ones, configuring a good starting point for deeper investigations. Moreover, pathways regulated by the miRNAs found were consistent with the exercise physiology background.

### 4.4. Perspectives

High-performance endurance athletes’ miRNA profiles could provide valuable insights for targeted, personalized, and optimized strategies based on individual biological factors. The miRNA profiles and their physiologic prediction effects can be used to monitor training progress and adaptations or even to predict performance and VO_2_max levels. Finally, our results indicate the possibility of nutrition adjustment based on lipid income and training levels regarding lipid expenditures. Starting from these valuable lipid-related results, a lipidomics analysis with the possibility of measuring the impact of all circulating lipids directly from the plasma is the natural follow-on from these findings.

## 5. Conclusions

Plasma detection and analysis of a miRNA panel were able to group unique distance runners according to a GXT and their performance, providing some miRNA-related candidates. Harmoniously to some physiological requirements of endurance performance, our results showed that circulating miRNAs related to fatty acid metabolism might contribute to sustaining high endurance performance and enable more efficient recovery time in elite athletes. In addition to fatty acid metabolism pathways, circulating miRNAs were important for essential amino acid metabolism, tissue repair via Hippo signaling pathways, immune response, and ECM remodeling via TGF-β and ECM–receptor pathways in response to endurance exercise and adaptation. Choosing a highly genetically dependent gold standard variable for evaluating endurance performance (VO_2_max) in a homogeneous group of elite athletes allowed genetic/epigenetic aspects to emerge, paving a scientific path for more profound targeted physiological and molecular interventions based on precision medicine.

## 6. Limitations

Updates in miRNA knowledge have provided corrected annotations for some RNAs previously annotated as miRNAs. Some were important for clustering our athletes, but they were not miRNAs and were not included in the FBA. This does not exclude the possibility that they could be important for the exercise field. The miRNA target databases like Tarbase, beyond the in silico prediction and some experimentally validated material, may contain some data derived from experimental cancer research, and this could partially limit the ability to analyze a wide range of miRNA roles in different biological processes. Furthermore, no mRNA transcriptomics or proteomic data were used to contribute to the validation of our miRNA targets. Although bioinformatics is a prominent and revolutionary field, our functional analysis is preliminary and conceptual, pointing to more profound, practical, targeted applications. Finally, while our initial findings with six unique individuals are promising, a larger sample size would allow for more robust conclusions and significance levels. Thus, to avoid the confounding variables cited for a bioinformatic approach, new studies should follow database updates, check the applicability of data available in systems biology, and look for validation of previous in silico indications using new methods.

## Figures and Tables

**Table 1:** Study population characteristics at baseline.

Physical-Related Variables	n = 6		*p*-Value
	HVO2 (n = 3)	LVO2 (n = 3)	
Age (years)	21.3 ± 3.5	26.6 ± 7.6	0.334
Weight (kg)	57.3 ± 1.5	66.8 ± 5.1	0.073
Height (cm)	175.3 ± 4.2	180.2 ± 3.0	0.172
Fat percentage	4.5 ± 0.3	5.9 ± 1.9	0.296
VO_2_max (mL.kg^−1^.min^−1^)	75.4 ± 0.9	60.1 ± 5.0	0.007 **
Weekly training volume (km)	130.0 ± 26.5	130.0 ± 26.5	0.999
Training experience (years)	5.0 ± 2.6	6.0 ± 6.9	0.827
IAAF points	725 ± 163	606 ± 101	0.340

Data are presented as mean and standard deviation. ** Significant difference (*p* ≤ 0.01). HVO2: higher maximum oxygen uptake (VO_2_max); LVO2: lower VO_2_max; IAAF: International Association of Athletics Federations.

**Table 2:** Comparison of the significant miRNAs for group comparisons. Statistical results are available in Tables S3 and S4 (supp. results).

miRNA	all_ PRE_vs_POST	Group Comparisons			
		HVO2 (PRE_vs_POST)	LVO2 (PRE_vs_POST)	PRE (HVO2_vs_LVO2)	POST (HVO2_vs_LVO2)
miR-1281	x	x	x	x	
miR-150-5p	x	x	x	x	
miR-26a-5p	x	x	x	x	
miR-4290	x	x	x	x	
miR-1308	x	x	x		
miR-154-5p	x	x	x		
miR-199b-3p	x	x	x		
miR-135b-5p	x	x		x	
miR-432-5p	x	x		x	
miR-219a-1-3p	x	x			x
miR-126-3p	x	x			
miR-3181	x	x			
miR-382-5p	x	x			
miR-486-5p	x	x			
miR-499a-3p	x	x			
miR-512-3p	x	x			
miR-92a-3p	x	x			
miR-10b-5p	x				
miR-1183	x				
miR-1260a	x				
miR-1273d	x				
miR-1292-5p	x				
miR-135a-5p	x				
miR-18b-5p	x				
miR-197-3p	x				
miR-1975	x				
miR-2110	x				
miR-223-3p	x				
miR-30d-5p	x				
miR-362-5p	x				
miR-4270	x				
miR-4286	x				
miR-4313	x				
miR-483-3p	x				
miR-500a-5p	x				
miR-548	x				
miR-571	x				
miR-766-3p	x				
miR-1826		x	x		x
miR-151a-5p		x		x	
miR-191-5p		x		x	
miR-23a-3p		x		x	
miR-92b-3p		x		x	
miR-1280		x			
miR-151-3p		x			
miR-1825		x			
miR-3138		x			
miR-3172		x			
miR-320a-3p		x			
miR-320e		x			
miR-323b-5p		x			
miR-449b-3p		x			
miR-572		x			
miR-320b			x		x
miR-132-3p			x		
miR-185-3p			x		
miR-494-3p			x		
miR-628-3p			x		
miR-1290				x	
miR-149-5p				x	
miR-195-5p				x	
miR-222-3p				x	
miR-1207-5p					x
miR-1229-3p					x
miR-125a-5p					x
miR-1538					x
miR-15a-5p					x
miR-1913					x
miR-1973					x
miR-370-3p					x
miR-612					x
miR-937-3p					x

**Table 3:** More conservative global pathways were obtained by up to 20 of the most significant key miRNAs in every comparison after Fishers’ exact test, false discovery ratio (FDR) correction, and additional EASE score, a correction tool for multiple comparisons for strict and more conservative statistics. *p*-values, the number of miRNAs involved, and their target genes are presented. Pathways highlighted in blue represent ECM-receptor interaction; in red, fatty acid biosynthesis; in orange, fatty acid metabolism; in purple, Hippo signaling; in gray, transforming growth factor (TGF-β); in yellow, lysine degradation; and in green, protein processes in endoplasmic reticulum (PPER) pathways. ECM: extracellular matrix; HVO2 = higher maximum oxygen uptake (VO_2_max); LVO2 = lower VO_2_max; POST = after exercise; PRE = before exercise.

**all_PRE_vs_POST**					**LVO2_PRE_vs_POST**					**HVO2_PRE_vs_POST**			
**KEGG Pathway**	***p*-Value**	**miRNAs**	**Genes**		**KEGG Pathway**	***p*-Value**	**miRNAs**	**Genes**		**KEGG Pathway**	***p*-Value**	**miRNAs**	**Genes**
Prion diseases	<1 × 10^−325^	1	1		Fatty acid biosynthesis	<1 × 10^−325^	2	1		Prion diseases	<1 × 10^−325^	2	9
ECM-receptor interaction	<1 × 10^−325^	3	11		ECM-receptor interaction	<1 × 10^−325^	2	6		ECM-receptor interaction	<1 × 10^−325^	3	11
Fatty acid biosynthesis	<1 × 10^−325^	4	4		Fatty acid metabolism	3.02 × 10^−7^	2	1		Lysine degradation	1.71 × 10^−5^	3	14
Fatty acid metabolism	<1 × 10^−325^	4	8		Hippo signaling pathway	1.85 × 10^−4^	3	41		Proteoglycans in cancer	1.55 × 10^−4^	4	59
					TGF-beta signal pathway	9.93 × 10^−4^	4	33		Fatty acid biosynthesis	3.68 × 10^−3^	1	1
										Adherens junction	6.66 × 10^−6^	5	34
**PRE_HVO2_vs_LVO2**					**POST_HVO2_vs_LVO2**					**all_4_groups**			
**KEGG Pathway**	***p*-Value**	**miRNAs**	**Genes**		**KEGG Pathway**	***p*-Value**	**miRNAs**	**Genes**		**KEGG Pathway**	***p*-Value**	**miRNAs**	**Genes**
ECM-receptor interaction	<1 × 10^−325^	1	7		Fatty acid biosynthesis	<1 × 10^−325^	2	4		Fatty acid biosynthesis	<1 × 10^−325^	3	4
Prion diseases	<1 × 10^−325^	2	9		Hippo signaling pathway	1.26 × 10^−12^	4	50		Fatty acid metabolism	<1 × 10^−325^	4	15
Proteoglycans in cancer	3.35 × 10^−8^	4	77		Fatty acid metabolism	9.50 × 10^−11^	2	15		ECM-receptor interaction	<1 × 10^−325^	3	11
Fatty acid biosynthesis	7.78 × 10^−5^	1	1		Adherens junction	1.24 × 10^−6^	5	36		Prion diseases	<1 × 10^−325^	1	1
Adherens junction	8.03 × 10^−5^	6	43		Viral carcinogenesis	7.91 × 10^−6^	3	70		Lysine degradation	1.09 × 10^−7^	3	18
Lysine degradation	1.38 × 10^−4^	3	12							Adherens junction	1.98 × 10^−3^	6	38
Arrhythmogenic right ventricular	6.42 × 10^−3^	2	8							Proteoglycans in cancer	5.03 × 10^−3^	4	57
Hippo signaling pathway	7.88 × 10^−3^	2	43							Viral carcinogenesis	3.35 × 10^−2^	4	78
Prot process in endop. reticulum	9.08 × 10^−3^	3	59										
TGF-beta signal pathway	1.13 × 10^−2^	4	34										
Viral carcinogenesis	1.68 × 10^−2^	3	71										
Pathways in cancer	3.20 × 10^−2^	3	131										

## Data Availability

Data, including raw RT-PCR and in silico functional analysis data, are freely available upon request.

## Supplementary Materials

The following supporting information can be downloaded at: https://www.mdpi.com/article/10.3390/genes15081088/s1, Supplementary methods and Supplementary results and Supplementary references. Figure S1: Schematic representation of subjects’ participation and blood samples obtained; Figure S2: Most stably expressed miRNAs across the dataset; Figure S3: All dataset Non-supervised Principal Component Analysis (PCA) graphs showing “timepoint” based clustering analysis between groups; Figure S4: Athletes participating in the study ranked from their decreasing levels of VO_2_max. In red HVO2 athletes. In blue, LVO2 athletes; Figure S5: Venn diagram regarding 20 key miRNAs for the clustering of every group; Figure S6: Fatty acid Biosynthesis pathway (hsa00061) from KEGG database; Table S1: Nomenclature of key miRNAs for every comparison; Table S2: Non-used RNA for miRNA functional bioinformatic analysis because they are not miRNA in the newest database version; Table S3: Up to 20 more significant miRNAs with log2Fold change differences < −1 or >1 for every comparison in Paired analysis; Table S4: Up to 20 more significant miRNAs with log2Fold change differences < −1 or >1 for every comparison in a Non-Paired analysis.
