# Supplementary material for: Circulating miRNA Signaling for Fatty Acid Metabolism in Response to a Maximum Endurance Test in Elite Long-Distance Runners
Source: Genes (Basel). 2024 Aug 17;15(8):1088. doi: 10.3390/genes15081088 (PMC11353293; doi:10.3390/genes15081088)
Supplement: Supplementary file 1 [file genes-15-01088-s001.zip › genes-3136280-supplementary.pdf]

SUPPLEMENTARY METHODS

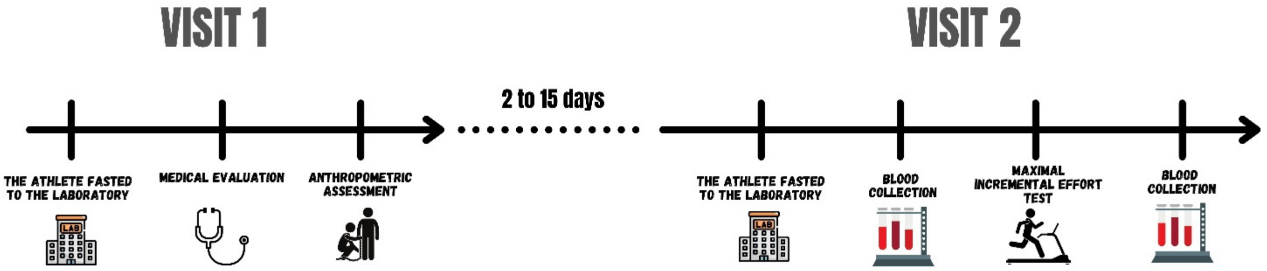

Fig.S1. Schematic representation of subjects' participation and blood samples obtained.

Anthropometric measurements

Participants were weighed using a mechanic balance (±0.1 kg resolution) (Welmy®). Height was measured with a stadiometer (±0.5 cm resolution) (Sanny®). Skinfold thickness was measured at eight body sites (i.e., triceps brachii, supra iliac, abdominal, chest, subscapular, midaxillary, mid-thigh and calf) using a plicometer with stem pressure of 10.0 g/mm² and scale resolution of 1.0 mm (Lange Skinfold Caliper®, Beta Technology®, Santa Cruz, CA, EUA). The median of the values was used for data analysis. The same investigator always performed measurements. Body fat was estimated using the equations of Jackson and Pollock.

Standardized Breakfast

One slice of bread, one banana, water

Ergometric protocol

Resting values for heart rate (HR) and minute ventilation (VE) were determined before maximal treadmill testing. Telemetry continuously monitored heart rate (HR) throughout the test (Polar S810 series®, Polar Electro Oy®, Kempele, Finland). VE was measured using a flow sensor with dynamic resistance. The fraction of expired oxygen was measured using a cold fuel cell system, while the fraction of carbon dioxide was measured using an infrared sensor, according to the manufacturer's specifications. The subjects used a silicone mask (V-Mask, Hans Rudolph Inc., Kansas City, MO, USA) attached to a turbine with a bidirectional gas flow ("MIR" Turbine, Vacumed, Ventura, CA, USA). VE, oxygen consumption per minute (VO_{2max}) and volume of carbon dioxide production (VCO_2) were sampled online and averaged every 30s through specific software (Vista Turbo Fit 5.1, Ventura, CA, EUA). Initially, the values were recorded for 6 minutes in the sitting position, and the mean value was calculated based on the average of the second to the fifth minute. After, a graded maximal and continuous exercise protocol was used to determine VO_{2max}. The test started at 14 $km.h^{-1}$, and the speed was increased by one $km.h^{-1}$ every 3 minutes, with 3 to 5 minutes of rest between each stage. The treadmill (Master Super ATL®, Inbramed®) inclination was set at a constant gradient of 5,25% for the entire test. Expired gases were analysed using open-circuit indirect calorimetry (Vista Mini-CPX, Vacumed, Ventura, CA, USA). The gas analyser was calibrated before each test with a certified gas mixture containing 16.0% oxygen and 4.0% carbon dioxide and balanced with nitrogen (Linde®). As a control, the fractions of oxygen and carbon dioxide were measured in the mixture used for calibration at the end of each test. In addition, every test was preceded by a one-point calibration to ambient air, whose partial O_2 composition was assumed to be 20.9% and gas of known CO_2

concentration (0.03%) (Vacumed instruction manual). Additionally, the calibration of the turbine flow meter of the Vacumed was performed with a 3L syringe before each test (Hans Rudolph®, EUA, Canada). The perceived exertion was determined using the Borg scale (6-20 point scale) in the last ten seconds of each stage. In this incremental protocol, VO2max was determined by at least three of the following test criteria: a) VO_2 plateau ≤ 150 $mL.min^{-1}$; b) respiratory exchange ratio (RER VCO_2/VO_2) ≥ 1.10; c) peak heart rate at least equal to 90% of the age-predicted; d) rating of perceived exertion ≥ 19.[1,2]

RT-PCR quality control

Additional manual careful validation for melting curves was performed. Cqs from dubious peaks or unspecific amplifications were not used. miRNAs with less than 33% of valid results were totally removed from the analysis. Cq higher than 37 were considered initially as low copy numbers of RNA. An extra penalty was applied in our data for the missing Cq values caused by the low amount of RNA. Missing Cq were replaced by the highest Cq value available for the gene in question, increased by two cycles. Interplate calibration was performed using U1 snRNA gene amplification, which is present in all plates. Best housekeeping genes were tested using NormFinder and geNorm tools in GenEx software v7.1.1.118. The geometrical mean of the ten best housekeeping candidates fitted better (lower variance) than the other genes or even the non-miRNA housekeeping genes suggested for the manufacturer protocol (Figure S2). Furthermore, the indicated genes are not miRNAs and did not represent similar behavior to plasma miRNAs.

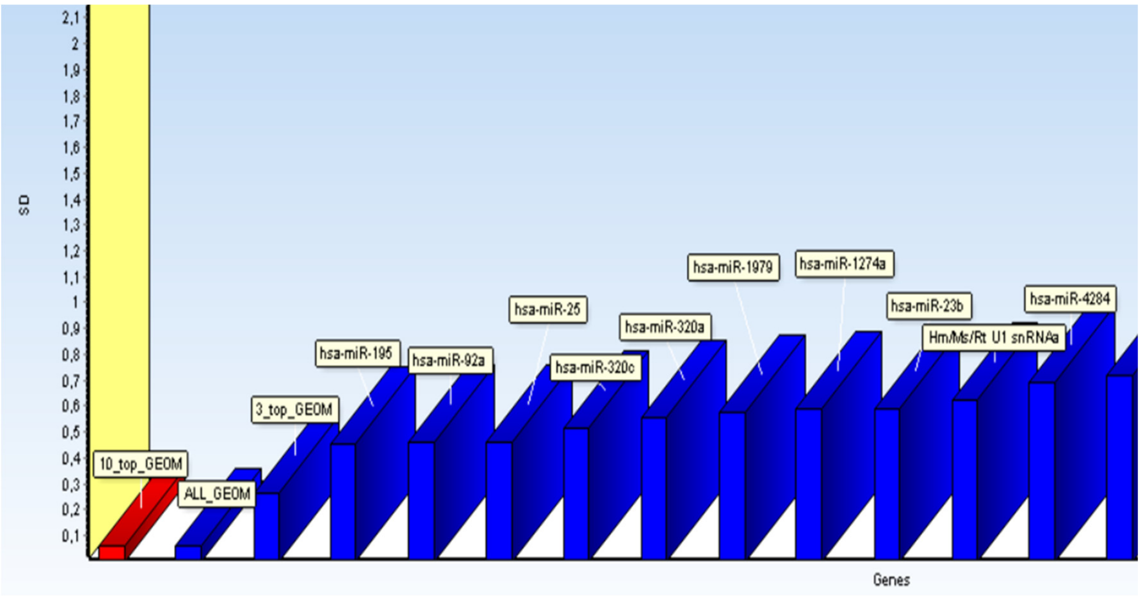

Fig.S2. Most stably expressed miRNAs across the dataset.

So, the resulting Cq values were normalised using the geometrical mean of the ten most stably expressed miRNAs across the dataset using the GenEx software v 7.1.1.118. The delta-Cq values were further processed to obtain the relative expression in relation to the sample with the lowest expression of each gene. Data were transformed using log_2 for parametric statistical analysis.

SUPPLEMENTARY RESULTS

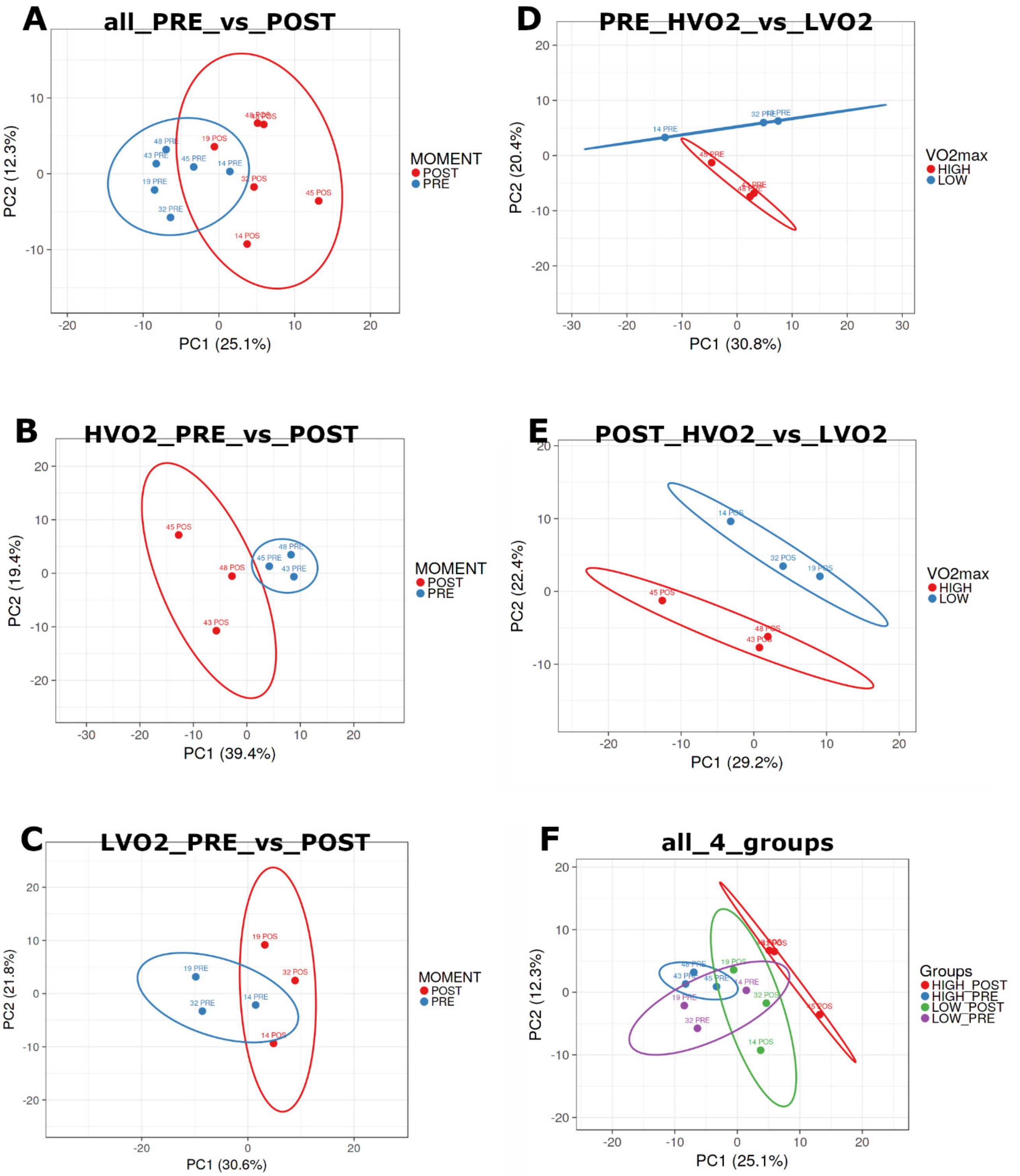

Fig.S3. All dataset Non-supervised Principal Component Analysis (PCA) graphs showing “timepoint” based clustering analysis between groups A: all_PRE_vs_POST, B: HVO2_PRE_vs_POST and C: LVO2_PRE_vs_POST comparisons; Additional PCA offering “performance” based clustering analysis between D: PRE_HVO2_vs_LVO2, E: POST_HVO2_vs_LVO2. And finally, a global clustering analysis between F: all_4_groups comparisons. PCAs showing partial clustering were performed using the relative amount of RNAs from the whole dataset. Confident intervals are presented inside every colored ellipse.

$\uparrow VO_2$máx

	$VO_{2máx}$(mL·kg·min^{-1})	$VO_{2máx}$(L·min^{-1})	Heart Rate $_{máx}$ (bpm)	$Vel_{máx}$(km·h^{-1})	$Vel_{máx}$(m·min^{-1})
High - $VO_{2máx}$					
Sujeito 1	78,9	5,23	176	21	350
Sujeito 2	78,2	5,18	189	20	333
Sujeito 3	76,1	4,47	186	20	333
Sujeito 4	76,0	4,48	195	19	317
Sujeito 5	75,8	4,25	191	18	300
Sujeito 6	75,4	5,67	180	19	317
Sujeito 7	75,2	4,36	192	17	283
Sujeito 8	74,4	4,22	185	18	300
Medium - $VO_{2máx}$					
Sujeito 9	71,7	3,93	197	19	317
Sujeito 10	71,5	5,19	207	18	300
Sujeito 11	70,8	4,83	187	18	300
Sujeito 12	69,8	4,79	196	19	317
Sujeito 13	69,3	4,25	194	18	300
Sujeito 14	68,6	4,53	187	18	300
Sujeito 15	68,1	4,63	183	17	283
Low - $VO_{2máx}$					
Sujeito 16	67,7	4,60	193	18	300
Sujeito 17	67,2	4,78	191	18	300
Sujeito 18	65,3	4,21	192	17	283
Sujeito 19	64,6	4,50	197	17	283
Sujeito 20	64,3	4,25	188	17	283
Sujeito 21	60,4	3,39	182	18	300
Sujeito 22	60,1	3,57	185	20	333
Sujeito 23	59,8	3,78	184	17	283
TOTAL_Average	70,0	4,48	189	18	305
SD	5,8	0,54	7	1	18

$\downarrow VO_2$máx

Fig.S4. Athletes participating in the study ranked from their decreasing levels of VO_2max. In red HVO2 athletes. In blue, LVO2 athletes

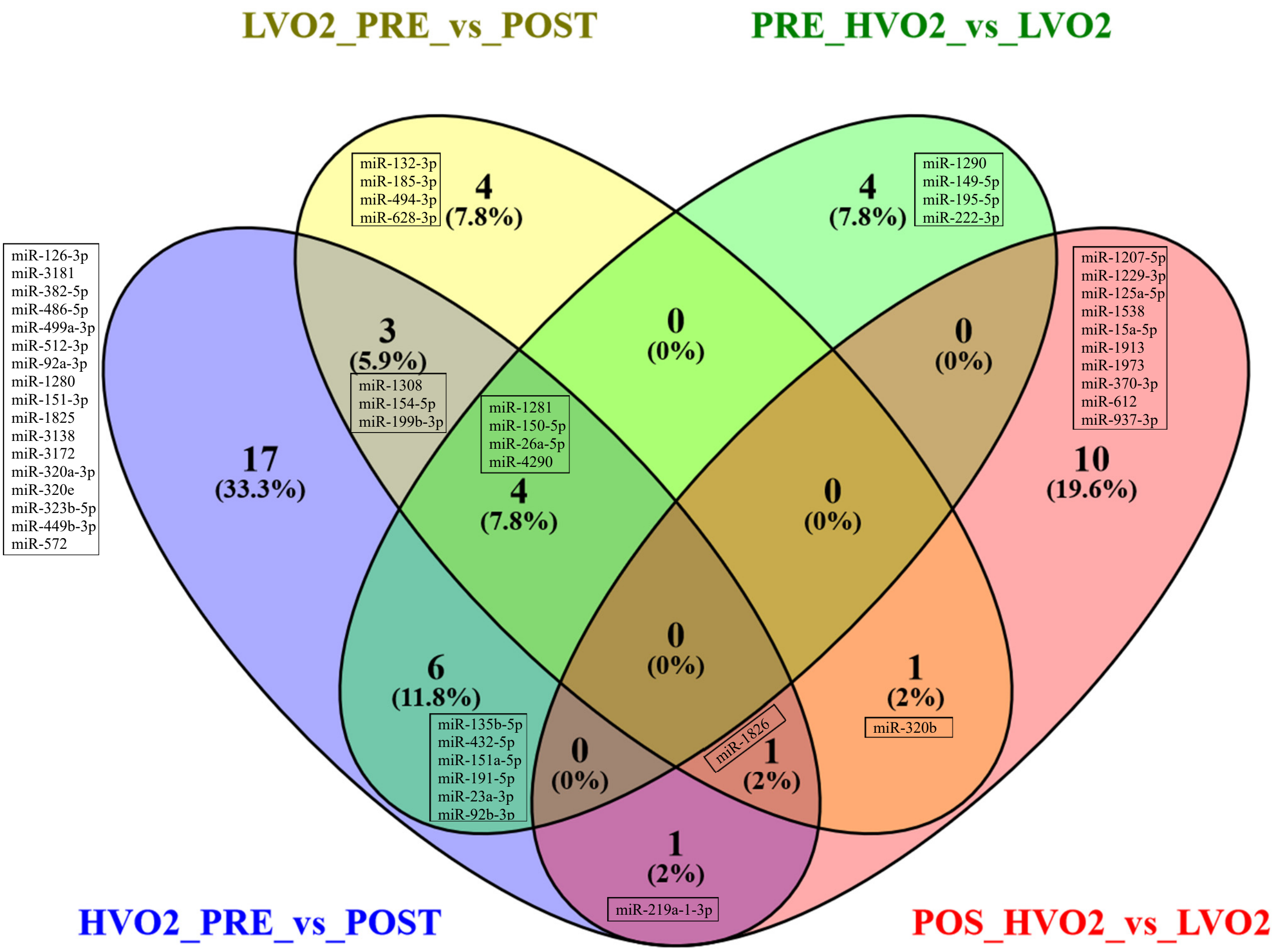

Fig.S5. Venn diagram regarding 20 key miRNAs for the clustering of every group. HVO2_PRE_vs_POST: most important miRNAs for separating PRE and POST exercise moments in the HVO2 group; LVO2_PRE_vs_POST: most important miRNAs for separating PRE and POST exercise moments in the LVO2 group; PRE_HVO2_vs_LVO2: most important miRNAs for separating the HVO2 and LVO2 at the PRE-exercise moment; POST_HVO2_vs_LVO2: most important miRNAs for separating the HVO2 and LVO2 at the POST-exercise moment.

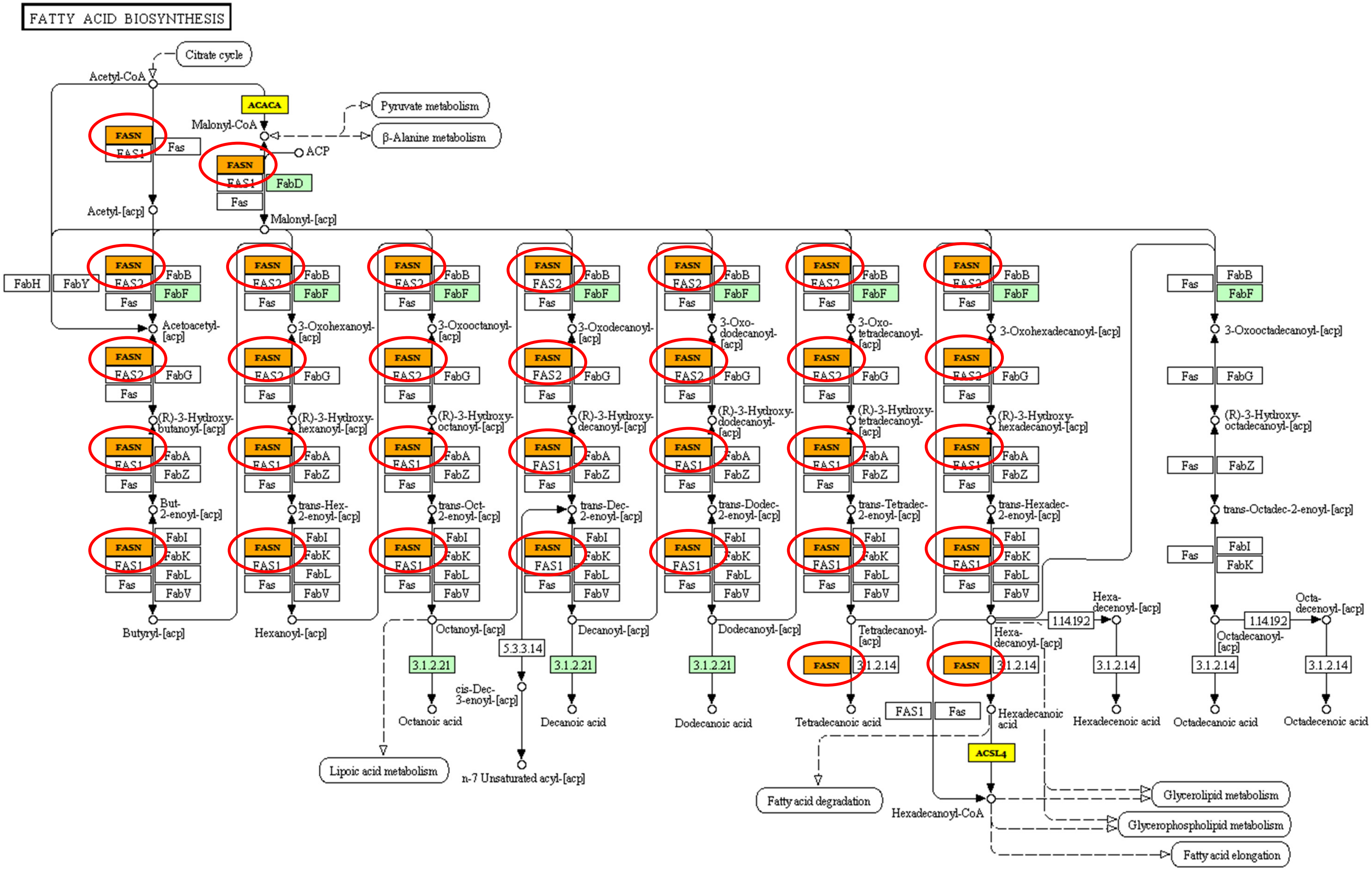

Fig.S6. Fatty acid Biosynthesis pathway (hsa00061) from KEGG database. *ACSL4*: Acyl-CoA synthetase 4. *ACACA*: Acetyl-CoA carboxylase alpha. *FASN*: Fatty acid Synthase. Red ellipses highlight multiple stages of *FASN* engagement.

Adjusted nomenclature for miRNAs.

The SYBR-based RT-PCR panel hsa-miRNome microRNA *Profiling Kit* (Human) from (System Biosciences, USA) is based on version 15 of miRbase. Using primers provided by the manufacturer, we updated the nomenclature following miRbase version 22.1 according to Table S1.

Table S1
Nomenclature of key miRNAs for every comparison, detected by the commercial kit according to version 15 and adjusted according to the most current version of the miRNAs database (miRBase).

Version 15	Version 22
hsa-miR-1292	hsa-miR-1292-5p
hsa-miR-1260	hsa-miR-1260a
hsa-miR-1229	hsa-miR-1229-3p
hsa-miR-1228	hsa-miR-1228-3p
hsa-miR-937	hsa-miR-937-3p
hsa-miR-766	hsa-miR-766-3p
hsa-miR-500	hsa-miR-500a-5p
hsa-miR-494	hsa-miR-494-3p
hsa-miR-451	hsa-miR-451a
hsa-miR-449b*	hsa-miR-449b-3p
hsa-miR-432	hsa-miR-432-5p
hsa-miR-382	hsa-miR-382-5p
hsa-miR-370	hsa-miR-370-3p
hsa-miR-323-5p	hsa-miR-323b-5p
hsa-miR-320a	hsa-miR-320a-3p
hsa-miR-223	hsa-miR-223-3p
hsa-miR-222	hsa-miR-222-3p
hsa-miR-219-1-3p	hsa-miR-219a-1-3p
hsa-miR-199b-3p	hsa-miR-199b-3p
hsa-miR-197	hsa-miR-197-3p
hsa-miR-195	hsa-miR-195-5p
hsa-miR-191	hsa-miR-191-5p
hsa-miR-185*	hsa-miR-185-3p
hsa-miR-181c	hsa-miR-181c-5p
hsa-miR-154	hsa-miR-154-5p
hsa-miR-151-5p	hsa-miR-151a-5p
hsa-miR-150	hsa-miR-150-5p
hsa-miR-149	hsa-miR-149-5p
hsa-miR-135a	hsa-miR-135a-5p
hsa-miR-135b	hsa-miR-135b-5p
hsa-miR-132	hsa-miR-132-3p
hsa-miR-126	hsa-miR-126-3p
hsa-miR-122	hsa-miR-122-5p
hsa-miR-93	hsa-miR-93-5p
hsa-miR-92a	hsa-miR-92a-3p
hsa-miR-92b	hsa-miR-92b-3p
hsa-miR-30d	hsa-miR-30d-5p
hsa-miR-26a	hsa-miR-26a-5p
hsa-miR-23a	hsa-miR-23a-3p
hsa-miR-18b	hsa-miR-18b-5p
hsa-miR-15a	hsa-miR-15a-5p
hsa-let-7i	hsa-let-7i-5p
hsa-let-7c	hsa-let-7c-5p

Moreover, some RNAs analyzed were removed on the newest miRbase version because they were re-annotated correctly. The following RNAs were not used for miRNA functional bioinformatic analysis because they are not miRNA in the latest database version (Table S2).

Table S2
Non-used RNA for miRNA functional bioinformatic analysis because they are not miRNA in the newest database version.

RNA detected	Access from miRBase	Information from Database
hsa-miR-1308	https://www.mirbase.org/cgi-bin/mirna_entry.pl?acc=MI0006441	The mature miR-1308 is a fragment of a tRNA
hsa-miR-1975	https://www.mirbase.org/cgi-bin/mirna_entry.pl?acc=MI0009985	The annotated mature sequence is a fragment of Y RNA
hsa-miR-3172	https://www.mirbase.org/cgi-bin/mirna_entry.pl?acc=MI0014203	The mature miR-3172 is a fragment of a predicted tRNA
hsa-miR-1826	https://www.mirbase.org/cgi-bin/mirna_entry.pl?acc=MI0008194	The annotated mature sequence is a fragment of 5.8S rRNA.
hsa-miR-720	https://www.mirbase.org/cgi-bin/mirna_entry.pl?acc=MI0006654	The sequence annotated as miR-720 is likely to be a fragment of a tRNA
hsa-miR-1977	https://www.mirbase.org/cgi-bin/mirna_entry.pl?acc=MI0009987	Maps to the Mt genome and overlaps a Mt tRNA sequence
hsa-miR-1280	https://www.mirbase.org/cgi-bin/mirna_entry.pl?acc=MI0006437	miR-1280 is likely to be a fragment of a tRNA
hsa-miR-1979	https://www.mirbase.org/cgi-bin/mirna_entry.pl?acc=MI0009989	The mature miR-1979 is a fragment of a Y RNA
hsa-miR-1274a	https://www.mirbase.org/cgi-bin/mirna_entry.pl?acc=MI0006410	The putative mature miR-1274 sequence is a fragment of a Lys tRNA
hsa-miR-1978	https://www.mirbase.org/cgi-bin/mirna_entry.pl?acc=MI0009988	mir-1978 maps to the Mt genome and overlaps a Mt tRNA sequence

Table S3

Up to 20 more significant miRNAs with log_2Fold change differences <-1 or >1 for every comparison in Paired analysis. Green - miRNA confirmed from Database (miRBase v22.1). Pink – essential for clustering but removed from the functional analysis because they are not miRNA. Bold Underline Left Aligned - "Upregulated" in the first group (Left). Non-Bold Right aligned - "Upregulated" in the second group (right). Light Blue – log_2Fold change differences between -1 and 1.

	all_PRE_vs_ all_POST (2-tailed paired t-test)			HVO2_PRE_vs_POST (2-tailed paired t-test)			LVO2_PRE_vs_POST (2-tailed paired t-test)		
	(Paired) **all_PRE** vs (all_POST)	log2 fold change Difference (PRE-POST)	*p*-value	(Paired) **HVO2_PRE** vs (HVO2_POST)	log2 fold change Difference (PRE-POST)	*p*-value	(Paired) **LVO2_PRE** vs (LVO2_POST)	log2 fold change Difference (PRE-POST)	*p*-value
Up to 20 best-ranked miRNA (p≤0.05 and log2fold differences ≤ -1 or ≥ 1)	**hsa-miR-1308**	1,4	1,2E-04	**hsa-miR-92a-3p**	1,0	4,7E-04	**hsa-miR-1308**	1,3	1,7E-03
	hsa-miR-26a-5p	-2,9	8,3E-04	**hsa-miR-486-5p**	1,9	4,1E-03	**hsa-miR-199b-3p**	1,6	2,1E-03
	**hsa-miR-486-5p**	1,5	9,0E-04	**hsa-miR-512-3p**	2,0	5,8E-03	hsa-miR-494-3p	-1,6	3,7E-03
	**hsa-miR-1281**	4,5	1,0E-03	**hsa-miR-449b-3p**	1,0	6,4E-03	**hsa-miR-185-3p**	1,1	6,7E-03
	hsa-miR-1975	-1,5	3,4E-03	hsa-miR-26a-5p	-3,5	1,7E-02	**hsa-miR-4290**	1,8	7,0E-03
	hsa-miR-150-5p	-1,2	5,5E-03	hsa-miR-151a-5p	-2,0	1,8E-02	**hsa-miR-628-3p**	1,5	7,4E-03
	hsa-miR-4286	-1,9	7,1E-03	**hsa-miR-4290**	5,5	1,9E-02	hsa-miR-1826	-2,5	1,0E-02
	**hsa-miR-135a-5p**	2,4	9,0E-03	**hsa-miR-3138**	2,2	2,0E-02	hsa-miR-26a-5p	-2,2	1,3E-02
	**hsa-miR-432-5p**	2,3	9,0E-03	**hsa-miR-1825**	6,8	2,2E-02	hsa-miR-150-5p	-1,6	1,4E-02
	**hsa-miR-362-5p**	1,4	9,5E-03	**hsa-miR-92b-3p**	5,8	2,3E-02	hsa-miR-320b	-1,4	1,7E-02
	**hsa-miR-4290**	3,6	9,7E-03	**hsa-miR-1281**	5,3	2,4E-02	**hsa-miR-154-5p**	1,4	1,7E-02
	**hsa-miR-766-3p**	1,6	9,9E-03	hsa-miR-191-5p	-2,6	2,5E-02	**hsa-miR-132-3p**	2,4	5,0E-02
	**hsa-miR-154-5p**	2,5	1,0E-02	**hsa-miR-1308**	1,5	3,0E-02	**hsa-miR-1281**	3,7	5,0E-02
	**hsa-miR-199b-3p**	1,0	1,1E-02	**hsa-miR-320e**	1,4	3,0E-02			
	hsa-miR-223-3p	-1,3	1,1E-02	**hsa-miR-135b-5p**	2,5	3,4E-02			
	**hsa-miR-2110**	1,0	1,3E-02	**hsa-miR-219a-1-3p**	3,7	3,4E-02			
	**hsa-miR-1183**	1,5	1,4E-02	**hsa-miR-154-5p**	3,7	3,7E-02			
	**hsa-miR-219a-1-3p**	3,1	1,5E-02	hsa-miR-23a-3p	-1,5	4,1E-02			
	**hsa-miR-135b-5p**	2,0	1,6E-02	**hsa-miR-323b-5p**	2,0	4,1E-02			
	**hsa-miR-382-5p**	1,5	1,6E-02	**hsa-miR-432-5p**	2,0	4,1E-02			
Not used for functional bioinformatics analysis (FBA)	**hsa-miR-92a-3p**	0,9	5,0E-03	hsa-miR-320a-3p	-0,3	0,00562			
	**hsa-miR-500a-5p**	2,4	1,78E-02	**hsa-miR-3172**	0,7	0,01510			
	**hsa-miR-18b-5p**	0,9	1,91E-02	hsa-miR-150-5p	-0,7	0,00842			
	hsa-miR-3181	-1,6	1,93E-02	**hsa-miR-199b-3p**	0,5	0,00983			
	hsa-miR-10b-5p	-2,1	1,96E-02	hsa-miR-126-3p	-0,7	0,01672			
	hsa-miR-126-3p	-1,0	2,05E-02	**hsa-miR-572**	2,0	0,040848336			
	**hsa-miR-571**	1,6	2,07E-02	**hsa-miR-1280**	2,0	0,040848336			
	**hsa-miR-499a-3p**	1,9	2,23E-02	**hsa-miR-1826**	2,0	0,040848336			
	**hsa-miR-512-3p**	1,6	2,57E-02	**hsa-miR-382-5p**	2,1	0,044370198			
	**hsa-miR-483-3p**	2,2	2,66E-02	**hsa-miR-151-3p**	-1,3	0,046738405			
	hsa-miR-1273d	-2,6	2,71E-02	**hsa-miR-499a-3p**	2,6	0,048078118			
	hsa-miR-1292-5p	-1,1	2,82E-02	**hsa-miR-3181**	-1,9	0,049968723			
	hsa-miR-30d-5p	-0,6	3,05E-02						
	hsa-miR-4270	-3,2	3,30E-02						
	**hsa-miR-548**	2,5	3,43E-02						
	**hsa-miR-4313**	0,7	3,49E-02						
	hsa-miR-1260a	-1,8	3,64E-02						
	**hsa-miR-197-3p**	1,3	3,71E-02						

Green - miRNA confirmed and renamed following the most recent version from Database (miRBase v22.1)

Pink - They are in our pannel but they were Removed from miRBase - Funtionally they are not miRNA (But still important to clustering)

Bold Underline Left Aligned miRNAs - "Upregulated" in first group **(Left) -** for every comparision

Non-Bold Right alined miRNAs - "Upregulated" in the second group (right) - for every comparision

Light Blue - Highlighted log2Fold change differences between -1 and 1

Table S4

Up to 20 more significant miRNAs with log_2Fold change differences <-1 or >1 for every comparison in a Non-Paired analysis. Green - miRNA confirmed from Database (miRBase v22.1). Pink – essential for clustering but removed from the functional analysis because they are not miRNA. Bold Underline Left Aligned - "Upregulated" in the first group (Left). Non-Bold Right aligned - "Upregulated" in the second group (right). Light Blue – log2Fold change differences between -1 and 1.

	PRE_HVO2_vs_LVO2 (2-tailed t-test)			POST_HVO2_vs_LVO2 (2-tailed t-test)			HVO2_PRE_vs HVO2_POST_vs LVO2_Pre_vs LVO2_POST ANOVA	
	PRE (Non_Paired) **(HVO2)** vs (LVO2)	log2 fold change Difference (HIGH-LOW)	*p*-value	POST (Non_Paired) **(HVO2)** vs (LVO2)	log2 fold change Difference (HIGH-LOW)	*p*-value	Anova (Non_Paired) all_4_groups	*p*-value
Up to 20 best-ranked miRNA (p≤0.05 and log2fold differences ≤ -1 or ≥ 1)	hsa-miR-151a-5p	-1,9	3,0E-04	hsa-miR-1826	-5,4	1,9E-03	hsa-miR-26a-5p	2,7E-05
	hsa-miR-195-5p	1,0	7,7E-04	hsa-miR-15a-5p	-4,5	7,4E-03	hsa-miR-151a-5p	1,4E-04
	hsa-miR-1290	-4,4	1,1E-03	hsa-miR-320b	-1,0	1,2E-02	hsa-miR-1826	1,1E-03
	hsa-miR-432-5p	-2,8	3,3E-03	hsa-miR-1538	-1,4	1,2E-02	hsa-miR-4290	1,2E-03
	**hsa-miR-4290**	3,0	1,0E-02	hsa-miR-1973	-5,5	1,5E-02	hsa-miR-432-5p	1,2E-03
	hsa-miR-191-5p	-1,4	1,2E-02	hsa-miR-612	-3,8	1,8E-02	hsa-miR-1281	1,3E-03
	**hsa-miR-92b-3p**	3,6	1,2E-02	**hsa-miR-1207-5p**	3,4	2,4E-02	*hsa-miR-195-5p*	3,2E-03
	hsa-miR-26a-5p	-1,6	2,2E-02	**hsa-miR-370-3p**	3,3	2,4E-02	hsa-miR-219a-1-3p	4,8E-03
	hsa-miR-23a-3p	-1,2	2,7E-02	hsa-miR-1229-3p	-3,4	3,4E-02	hsa-miR-1973	5,4E-03
	**hsa-miR-135b-5p**	2,2	4,1E-02	hsa-miR-1913	-4,1	3,4E-02	hsa-miR-199b-3p	5,4E-03
	**hsa-miR-1281**	1,1	4,3E-02	**hsa-miR-125a-5p**	2,3	3,7E-02	hsa-miR-92a-3p	6,0E-03
	**hsa-miR-150-5p**	1,2	4,8E-02	hsa-miR-937-3p	-2,6	3,8E-02	hsa-miR-135b-5p	7,3E-03
	hsa-miR-222-3p	-1,0	4,9E-02	hsa-miR-219a-1-3p	-2,2	4,9E-02	hsa-miR-1228-3p	9,0E-03
	hsa-miR-149-5p	-1,0	5,0E-02				hsa-miR-382-5p	9,4E-03
							hsa-miR-4286	1,0E-02
							hsa-miR-92b-3p	1,1E-02
							hsa-miR-766-3p	1,3E-02
							hsa-miR-150-5p	1,4E-02
							hsa-miR-486-5p	1,4E-02
							hsa-miR-154-5p	1,5E-02
Not used for functional bioinformatics analysis (FBA)	**hsa-miR-126-3p**	0,8	2,7E-02				hsa-miR-320b	1,6E-02
							hsa-miR-612	1,7E-02
							hsa-miR-1207-5p	2,0E-02
							hsa-miR-197-3p	2,4E-02
							hsa-miR-1975	2,4E-02
							hsa-miR-10b-5p	2,5E-02
							hsa-miR-15a-5p	2,5E-02
							hsa-miR-1229-3p	2,8E-02
							hsa-miR-126-3p	3,0E-02
							hsa-miR-937-3p	3,0E-02
							hsa-miR-1290	3,2E-02
							hsa-miR-512-3p	3,3E-02
							hsa-miR-3181	3,7E-02
							hsa-miR-1979	3,8E-02
							hsa-miR-362-5p	4,1E-02
							hsa-miR-499a-3p	4,2E-02
							hsa-miR-3154	4,8E-02
							hsa-miR-500a-5p	5,0E-02

Green - miRNA confirmed and renamed following the most recent version from Database (miRBase v22.1)

Pink - They are in our pannel but they were Removed from miRBase - Funtionally they are not miRNA (But still important to clustering)

Bold Underline Left Aligned miRNAs - "Upregulated" in first group (Left) - for every comparision

Non-Bold Right alined miRNAs - "Upregulated" in the second group (right) - for every comparision

Light Blue - Highlighted log2Fold change differences between -1 and 1

SUPPLEMENTARY REFERENCES

1. Howley ET, Bassett DR, Welch HG. Criteria for maximal oxygen uptake. *Med Sci Sports Exerc*. 1995;27(9):1292???1301. doi:10.1249/00005768-199509000-00009
2. Åstrand PO. Quantification of exercise capability and evaluation of physical capacity in man. *Prog Cardiovasc Dis*. 1976;19(1):51-67. doi:10.1016/0033-0620(76)90008-6
